# Recent Advances in Exploring Casein Peptide Regulation of Inflammatory Bowel Disease from an Intestinal Barrier Perspective: Correlations, Mechanisms, Challenges and Solutions

**DOI:** 10.3390/foods15060997

**Published:** 2026-03-11

**Authors:** Tingting Dong, Jiahui Ye, Jinquan Zhang, Wanxuan Song, Shuaibo Xia, Xinyan Li, Mengyao Liu, Daodong Pan, Zhen Wu, Maolin Tu

**Affiliations:** 1 State Key Laboratory for Quality and Safety of Agro-Products, Zhejiang Key Laboratory of Food Microbiology and Nutritional Health, Zhejiang-Malaysia Joint Research Laboratory for Agricultural Product Processing and Nutrition, College of Food Science and Engineering, Ningbo University, Ningbo 315211, China; 2 State Key Laboratory of Geriatric Nutrition and Health, Beijing Technology and Business University, Ministry of Education, Beijing 100048, China

**Keywords:** casein peptides, inflammatory bowel disease, intestinal barrier, mechanisms

## Abstract

Inflammatory bowel disease (IBD) is characterized by chronic and intermittent symptoms, exerting a profound impact on overall health. Although drug therapy and biological agents are primary treatment approaches for IBD, the side effects can affect human health. Thus, it is an urgent need to explore new approaches to counteract the harm caused by IBD. Owing to their natural origin and excellent biosafety, casein peptides are a promising candidate treatment for IBD. This review systematically outlines the structural basis of the intestinal barrier and elucidates the pivotal role of barrier dysfunction in IBD pathogenesis. We further elaborate on the multi-faceted therapeutic mechanisms of casein peptides in IBD, including intestinal barrier repair, immune homeostasis modulation, inflammatory response suppression, and other such pathways. Moreover, we analyze the key challenges of intestinal-barrier-targeted casein peptide therapies in current research and translational practice, and propose future perspectives for overcoming these limitations, thus providing a reference for potential new preventive and therapeutic approaches to IBD.

## 1. Introduction

Inflammatory bowel disease (IBD) is a recurrent inflammation in the gut lumen and has already become a global problem. It seriously affects quality of life and imposes a substantial socioeconomic burden [1]. Over the last decade, the morbidity rate of IBD has increased globally, and the affected population is gradually becoming younger. Although gastroenterologists in some countries can access new biological agents and small molecules, most IBD patients worldwide do not have easy access to anti-tumor necrosis factor (TNF) biological agents [2].

A growing body of clinical evidence has firmly established the association between intestinal barrier dysfunction and IBD. The primary objective of treating IBD is to restore the integrity of the intestinal barrier, as this constitutes the core mechanism of the disease [3]. As a vital protective system, the intestinal barrier comprises three layers. This complex structure within the human digestive system not only absorbs nutrients but also blocks pathogens and toxins while ensuring internal balance [4]. Its outermost mucus layer hosts commensal microbiota and contains antimicrobial compounds. The middle layer consists of a continuous epithelium of intestinal cells. The innermost stratum, known as the lamina propria, is populated by immune cells that coordinate both innate and adaptive immune responses [5]. Damage to the intestinal barrier may trigger various diseases including IBD, celiac disease, irritable bowel syndrome, colorectal cancer, type 1 diabetes, obesity, and neurodegenerative diseases. Compared with new biological agents, small-molecule drugs have good oral bioavailability for treating diseases caused by intestinal damage, but they still have side effects such as upper respiratory tract infections, bronchitis, nausea and cough [6]. Worsening health from drugs use imposes significant costs on healthcare systems. Consequently, there is a critical need to develop novel therapeutic agents for IBD management.

Bioactive peptides, in addition to their fundamental nutritional value, can also serve as potential modulators for disease prevention. For this reason, bioactive peptides have the potential to reduce patients’ dependence on medications and lower healthcare costs [7]. Bioactive peptides may be categorized according to their source, such as peptides derived from animal, plant, or marine proteins [8]. Animal-derived bioactive peptides are primarily obtained from milk [9], meat, and egg [10] proteins. Plant-derived bioactive peptides include those from soybeans [11] and flaxseed. Marine bioactive peptides are typically extracted from aquatic organisms such as fish and shellfish. Food-derived bioactive peptides demonstrate high safety, wide availability, and efficient digestion and absorption [12]. At the same time, compared to conventional pharmaceuticals, bioactive peptides derived from foodstuffs offer a natural approach to treating intestinal disorders with fewer side effects [13]. Milk contains a variety of active beneficial components that help maintain the integrity and stability of the intestinal barrier structure [14]. In addition, milk protein is regarded as a key provider of bioactive peptides [15]. In particular, when compared to other dietary sources of bioactive peptides, whey protein and casein—two key components of milk protein–contain essential amino acids and act as crucial substrates for peptide production. Milk protein contains approximately 80% casein, which makes it an important source of bioactive peptides [16]. Consequently, casein peptides possess the potential to regulate intestinal barrier function, offering promising support for alleviating IBD.

In recent years, modulating intestinal barrier function has emerged as an important research direction for the alleviation of IBD. However, the role of casein peptides in this field has not yet been systematically summarized. Accordingly, this paper systematically reviews relevant research based on database retrieval. The retrieval methods are as follows: Literature searches were performed using the PubMed, Web of Science, and Scopus databases with the keywords “casein peptides”, “intestinal barrier”, and “IBD”. During the literature-screening process, inclusion criteria were primarily studies related to the regulation of the intestinal barrier by casein peptides; dairy-related publications irrelevant to the research topic were treated as exclusion criteria. This article discusses the relationship between intestinal barrier function and IBD, elucidates the mechanisms by which casein peptides regulate the intestinal barrier function, and proposes their barrier-repairing effect via a bidirectional gut–brain axis system. It also proposed the challenges and solutions of casein peptides, aiming to provide more effective approaches in the therapy of IBD.

## 2. A Compromised Intestinal Barrier: A Bridging Pathogenesis in IBD

**Figure 1:**
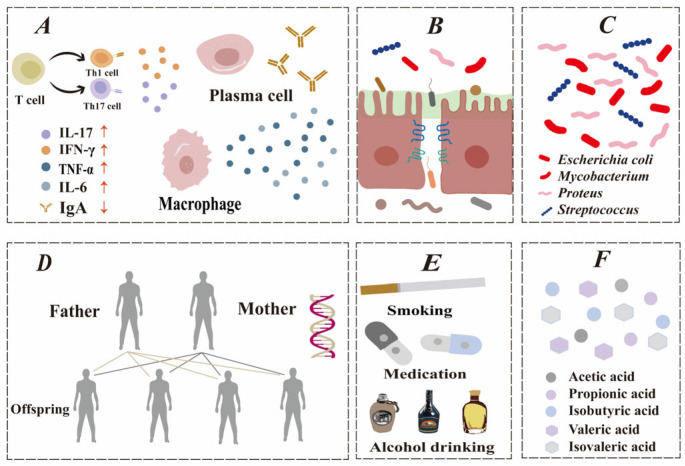
Factors involved in the pathogenesis of IBD. (**A**) Immune system disorder. (**B**) Intestinal barrier dysfunction. (**C**) Intestinal microbiota alteration. (**D**) Genetic factors. (**E**) Environmental factors (examples). (**F**) Metabolites.

Casein peptides help maintain intestinal barrier integrity through multiple biological pathways. Among these pathways, barrier function is a key factor relevant to IBD. Although IBD etiology remains incompletely defined, current research identifies that the primary factors influencing IBD include immune abnormalities, genetic susceptibility, gut microbiota [17], and other potential factors [18], as shown in Figure 1. The deterioration of the gut barrier is a key initial step in IBD, creating a continuous inflammatory cycle that barrier-targeting therapies can mitigate.

### 2.1. Impaired Intestinal Barrier Is an Important Cause of IBD

IBD refers to chronic gut disorders, including ulcerative colitis (UC) and Crohn’s disease (CD), whose origins are not fully known. UC, in particular, is characterized by persistent inflammation localized to the colorectal mucosa [19]. UC causes ongoing diarrhea, abdominal pain, and rectal bleeding from inflammation in the large bowel’s surface lining. CD can affect any gut area with deep inflammation, leading to intestinal narrowing, abnormal tunnels, and body-wide symptoms [20]. IBD is understood to result from a combination of environmental triggers, genetic predisposition, and abnormal immune activity [21], and intestinal barrier damage is a key factor in the pathogenesis of IBD. A study following 1420 healthy family members of Crohn’s patients demonstrated that elevated gut permeability predicts disease development, supporting barrier function evaluation for early intervention [22]. Studies indicate that intestinal barrier breakdown in IBD leads to interferon-gamma and TNF-alpha release, which causes ongoing inflammatory responses [23]. Another study found that imbalance between obligate and facultative anaerobes is a form of gut dysbiosis linked to IBD progression [24]. Consequently, intestinal barrier dysfunction is considered one of the primary causes of IBD pathogenesis.

### 2.2. IBD Will Further Damage the Intestinal Barrier

Intestinal barrier dysfunction plays a central role in the pathogenesis of IBD, and IBD can aggravate barrier dysfunction. The intestinal barrier of patients is damaged to varying degrees, including alterations in the mucus layer, shifts in the gut microbiota composition, and heightened epithelial permeability. Barrier function was evaluated by intraperitoneal injection of thymosin β4 (Tβ4) in C57BL/6 mice. Experimental data revealed the colitis mouse model had decreased levels of mucin 2 (MUC2) and increased Tβ4 expression. It was further shown that Tβ4 inhibited the production of colonic MUC2 and disrupted tight-junctions (TJs), leading to damage of the mucus barrier [25].

Gut microbiota analysis was performed on 313 individuals with IBD and 582 healthy subjects via 16S rRNA gene sequencing [26]. The results demonstrated a significant shift in the microbial community in the IBD group compared to healthy subjects. Specifically, the IBD group exhibited reduced levels of *Firmicutes* and *Actinobacteria*, but an elevated abundance of *Bacteroidetes.* Assessment via the lactulose–mannitol ratio (LMR) demonstrates persistently elevated intestinal permeability in IBD patients [27]. In the 24 h assessment protocol, researchers administered lactulose (1000 mg) and mannitol (100 mg) orally to subjects and measured urinary recovery rates at three designated time points to evaluate intestinal permeability [28]. Disease activity was simultaneously recorded using standardized endoscopic criteria. Results based on LMR revealed persistently elevated intestinal permeability in IBD patients, with LMR remaining at high levels across all disease stages. Comparative analysis demonstrated that intestinal permeability measurements in IBD patients were significantly higher than in healthy controls, and this difference remained statistically significant even during clinical remission. In conclusion, IBD can lead to dysfunction of the intestinal barrier in patients.

### 2.3. Repairing the Intestinal Barrier May Improve IBD

**Figure 2:**
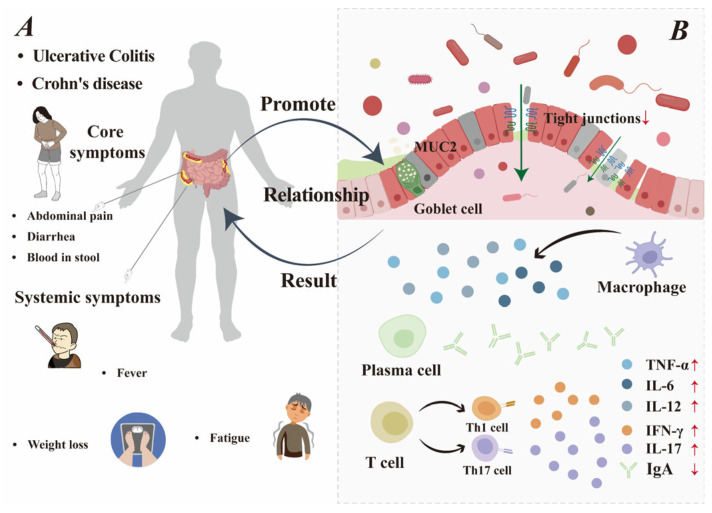
The relationship between IBD and impaired intestinal barrier. (**A**) Patients with IBD: core symptoms and systemic symptoms. (**B**) Impaired intestinal barrier.

Preserving a healthy intestinal barrier represents a central goal in the clinical management of IBD [29], and IBD can also be improved by strengthening the repair of gut barrier structural competence. The details of this relationship are shown in Figure 2. Two novel peptides from *Bombyx mori* demonstrating multiple beneficial effects in vitro, including alleviating oxidative stress, enhancing microbial diversity, promoting short-chain fatty acid production, and strengthening intestinal barrier function [30]. Glycomacropeptide (GMP) regulates the gut microbiota by promoting the growth of beneficial lactic acid bacteria and inhibiting *Enterobacteriaceae*, thereby alleviating intestinal dysfunction and improving overall health [31]. Casein glycomacropeptide significantly reduces the expression of cell adhesion molecules, markedly stimulating secretory immunoglobulin A (sIgA) secretion to enhance intestinal immunity while directly inhibiting the MAPK pathway and activating the transforming growth factor-β1 (TGF-β1)/Smad signaling cascade. This maintains the immunoregulatory function of the intestinal mucosa and protects its barrier integrity [32]. Consequently, by facilitating the repair of the intestinal barrier, casein peptides thereby could alleviate the symptoms of IBD.

## 3. The Regulatory Mechanisms of Casein Peptides on Intestinal Barrier Function

**Figure 3:**
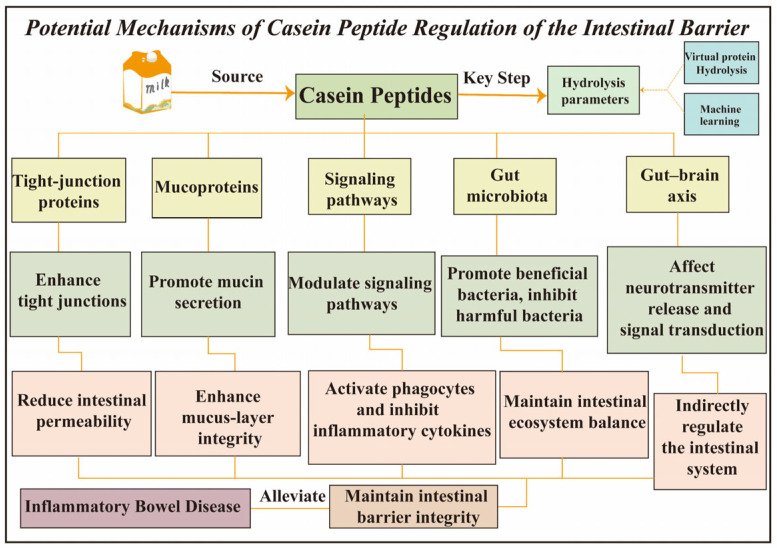
Potential mechanisms of casein peptide regulation of the intestinal barrier.

The key step in obtaining bioactive peptides with the desired properties lies in the determination of hydrolysis parameters, which can be identified by an innovative screening platform that consists of virtual protein hydrolysis and machine learning techniques [33]. As can be seen from Table 1, bioactive milk peptides have anti-inflammatory and immunoregulatory functions [34]. Casein peptides may safeguard intestinal barrier function through five distinct mechanisms, as in Figure 3: (1) enhancing intercellular bonds to limit permeability and block pathogens; (2) stimulating mucin production to maintain mucus barrier integrity; (3) regulating immune signals to suppress inflammation and activate macrophages; (4) promoting beneficial gut microbiota while inhibiting harmful strains; and (5) modulating gut–brain axis communication to indirectly support intestinal equilibrium.

### 3.1. Fundamentals of Structure–Activity Relationships of Casein Bioactive Peptides

Peptides derived from milk casein degradation generally fall below 6000 Da, with the major fraction consisting of 2–20 amino acid residues [46]. Low-molecular-weight peptides are more readily absorbed due to their smaller size. The functional properties of peptides are influenced by structural factors such as molecular weight, peptide chain length, amino acid sequence, charge, and hydrophobicity [47]. The anti-inflammatory activity of casein peptides is closely associated with specific amino acid compositions. Bioactive peptides with anti-inflammatory activity feature N-termini rich in basic amino acids such as lysine, arginine, and histidine, while their C-termini predominantly contain polar amino acids. Most peptides exhibit a net charge under physiological pH conditions [48]. Casein peptides exhibit N-terminal basic and C-terminal polar amino acid distributions that are associated with anti-inflammatory activity, which may provide the structural basis for their intestinal anti-inflammatory functions [46]. Zhao et al. [38] identified two hydrophobic anti-inflammatory peptides, DQPFFHYN and YSPFSSFPR, with YSPFSSFPR possessing an arginine residue at its C-terminus. Both peptides significantly inhibited the production of nitric oxide, TNF-α, IL-6 and iNOS.

### 3.2. Casein Peptide-Mediated Modulation Pathways of Intestinal Barrier Based on Structure–Activity Relationship

#### 3.2.1. Boosting TJ Proteins Levels Strengthens the Intestinal Barrier

The gut’s physical barrier is built from mucosal epithelium, cellular junctions, and the basement membrane. This barrier system not only facilitates the absorption of essential nutrients and fluids, but also effectively blocks the passage of pathogenic microorganisms [49]. Intercellular junctions primarily consist of TJs, adhesion junctions (AJs), and desmosome, among which TJs are paramount for maintaining barrier integrity [50]. TJs are mainly composed of key transmembrane proteins such as occludin and claudin, which are linked to the actin cytoskeleton by cytoplasmic plaque proteins like ZO-1 [51]. The integrity of epithelial structures is regulated by the actin-myosin cytoskeleton, which also serves as a key regulator of intercellular junctions and cell-matrix adhesion. The junctional belt, composed of actin and non-actin-binding myosin, maintains the integrity of TJs and AJs along with the paracellular barrier function. In contrast, actin-mediated focal adhesions primarily regulate cell-extracellular matrix interactions, cell polarity, and cell migration [52]. Multiple proteins including myosin light-chain kinase (MLCK), phospholipase C, protein kinase A, and mitogen-activated protein kinase regulate TJs, with MLCK playing a key role [53]. The inflammatory response activates myostatin-like protein kinase, which promotes MLCK expression and increases myosin light-chain phosphorylation. This induces actin-myosin ring contraction and structural rearrangement of TJ proteins, leading to increased intestinal barrier permeability [54]. Intestinal permeability is a quantitative parameter, it measures the speed at which paracellular and transcellular pathways traverse the monolayer of solutes in the epithelium [55].

Following the induction of colitis with dextran sulfate sodium (DSS), mouse colon tissue exhibited substantially lowered levels of major TJ proteins, while this effect was contrasted by a significant recovery in ZO-1, claudin-1, and occludin following administration of protein-sourced peptides [30]. The research team measured mRNA changes in TJ-related genes within Caco-2 cells treated with the casein peptide Asn-Pro-Trp-Asp-Gln and confirmed corresponding protein levels by immunoblotting. Finally, they found a specific upregulation of occludin with no significant effect on claudin-1 or ZO-1 [56]. Studies show that MAMP-1 alleviates intestinal injury in NEC murine models by upregulating ZO-1 to strengthen the intestinal barrier and reduce epithelial cell apoptosis to preserve integrity [57]. Therefore, casein peptides could improve intestinal barrier integrity and reduce permeability by enhancing the expression of TJ proteins.

#### 3.2.2. Increased Mucin Production and Enhanced Mucus Integrity

The intestinal mucus layer secreted by goblet cells primarily consists of MUC2, a heavily glycosylated protein with gel-forming properties [58], which integrates antimicrobial agents from paneth and enterocytes. This layer exhibits a bipartite structure in the colon: a dense inner stratum and a loose outer stratum. The latter comprises a mixture of MUC2, sIgA, and a spectrum of lamina propria-synthesized antimicrobial peptides, including defensins generated during adaptive immunity. The internal layer and the densely packed layer (non-stirred mucus layer) are firmly adhered to the monolayer of epithelium and are impermeable to microorganisms. Structurally, MUC2 exhibits significant homology with the adhesins utilized by bacteria for surface attachment. By occupying the critical adhesion sites, it prevents bacterial attachment to the gut epithelium, leading to bacterial retention in the mucous membrane layer and eventual expulsion through peristaltic movement [59]. A decrease in MUC2 content leads to damage of the mucus barrier, thereby causing UC [60].

Zhou et al. [30] confirmed through AB/PAS and MUC2 immunohistochemical staining that PDLGLF and GTEGGFPF treatments significantly restored the diminished colonic goblet cell numbers, mucus content, and MUC2 protein levels in DSS-induced colitis mice. In vitro studies have demonstrated that β-CN (94–123), a peptide derived from β-casein of yogurt, can upregulate the secretion of MUC2 and the expression of transmembrane MUC4 in human intestinal HT29-MTX goblet cells, which contributes to the maintenance or restoration of intestinal homeostasis and plays a crucial role in defending against harmful substances in the intestinal lumen [61]. Thus, casein peptides prompt increased MUC2 generation for a fortified mucus barrier that effectively blocks bacterial invasion and safeguards intestinal health.

#### 3.2.3. Regulation of Key Signaling Pathways and Inflammatory Mediators Production

The intestinal immune barrier is a sophisticated protective system built from gut-associated lymphoid tissue (GALT), diverse immune cells and sIgA. GALT maintains foundational immune monitoring in the gut lumen by establishing key tolerance processes [62]. Through its identification of bacterial antigens, sIgA builds an immune shield that stops pathogens from settling in the gut lining. Macrophages and natural killer cells are central to innate immunity in the lamina propria, establishing crucial gut protection and executing specialized immune activities [59]. GMP derived from κ-casein demonstrates compelling anti-inflammatory activity through its 35% suppression of nitrite and IL-1β alongside a 49% reduction in TNF-α production in U937 macrophage studies [63]. GMP achieves integrated immunomodulation in UC mice not only through simultaneously reducing intestinal CD4, CD8 and MAdCAM-1 levels, but also increasing secretory sIgA to enhance mucosal barrier protection [32].

The integrity of the gut barrier is coordinately regulated by multiple signaling pathways, among which TLR-mediated immune responses and downstream pathways such as MAPK/MLCK and PI3K/AKT play critical roles in regulating inflammatory responses and maintaining intestinal barrier function. GMP inhibits the NF-κB pathway, reducing IL-8 and TNF-α, thereby preventing activation of the MLCK pathway and maintaining the integrity of the intestinal barrier [64]. Li et al. [65] found that the immunomodulatory casein peptide SPAQILQW binds to TLR2 and TLR4, triggering signal cascade amplification and activating the MAPK signaling pathway, thereby regulating immune cell function and maintaining intestinal barrier homeostasis. Complementary Western blotting and bioinformatics analysis identified that the casein derived peptide YFYPEL counteracts intestinal epithelial cell dysfunction. The underlying mechanism involves the regulation of the PI3K/AKT pathway, leading to suppressed inflammatory cytokine production and promoted cell migration [66]. Current research findings confirm that casein peptides mitigate intestinal barrier damage through mechanisms involving the regulation of signaling pathways and the inhibition of inflammatory mediator release.

#### 3.2.4. Modify the Makeup of Intestinal Flora

The gut harbors a vast microbial ecosystem that significantly impacts human health. The intestinal microbiota ecosystem is a source of various bioactive substances, such as bacterial proteases. Proteases primarily cleave the peptide bonds linking adjacent amino acid residues in protein molecules, generating shorter peptides and amino acids [67]. The in situ production of peptides in the gut promotes the stability of the intestinal microbiota and maintains the integrity of the intestinal barrier [68]. Bacterial proteases maintain the intestinal barrier under controlled conditions by regulating TJ proteins and reducing intestinal permeability; however, excessive release of bacterial proteases can cause inflammatory diseases [69]. Beneficial microorganisms in the intestine can produce protease inhibitors that suppress excessive protease growth, potentially exerting positive effects on intestinal inflammation [70]. For example, *bifidobacteria* produce serine protease inhibitors that stimulate proteins in potential pathogenic bacteria, thereby exerting corresponding beneficial effects on the colon [71]. Accordingly, the composition of gut microbiota plays a crucial role in intestinal health.

The gut microbiota aid in enhancing the host’s ability to extract energy from food; their metabolic products and by-products can promote intestinal peristalsis and energy consumption [72]. Beyond forming a protective bacterial biofilm through adhesion to the intestinal epithelial mucosa, gut microbiota enhance barrier function by promoting the synthesis of TJ proteins, enhancing the mucosal layer and stimulating sIgA secretion [73]. The intestinal microbiota of healthy adults are mainly dominated by Gram-positive *Firmicutes* and Gram-negative *Bacteroidetes*, which account for 90% of the intestinal flora, followed by *Proteobacteria*, *Verrucomicrobiota*, and *Actinomycetes* [74]. The *Firmicutes*-to-*Bacteroidetes* (F/B) ratio is widely used to assess gut microbiota changes. Furthermore, a metagenomic analysis revealed 43 *Firmicutes* ribotypes in healthy individuals (n = 6) and 13 ribotypes in CD patients (n = 6), demonstrating a marked reduction in the phylogenetic richness of this major phylum in CD [75].

Li et al. [35] conducted a 8-week double-blind controlled trial and showed that casein peptides inhibit ACE to activate butyrate and propionate production, while increasing anti-inflammatory microorganisms. Casein hydrolysate raised the F/B ratio and *Eubacterium* levels to enhance microbial equilibrium [76]. These findings collectively show that casein peptides contribute to an improved gut microbiota profile with a higher F/B ratio, thereby promoting microbial balance and enhancing intestinal barrier integrity.

#### 3.2.5. Gut–Brain Axis

Functional coordination between the brain and gut is orchestrated by an integrated neural endocrine and immune signaling system [77]. The gut–brain axis enables two-way signaling between intestinal microbiota and the brain, which is key to sustaining balance across the digestive, nervous, and microbial environments [78]. Gut–brain axis signaling depends on vagus nerve transmission of neuro-immune information through the parasympathetic system, and the integrity of its functional pathway is crucial for maintaining the intestinal barrier [79]. The vagus nerve senses gut microbiota and their metabolites through its peripheral endings, relaying this information directly to the central nervous system (CNS) [80].

**Figure 4:**
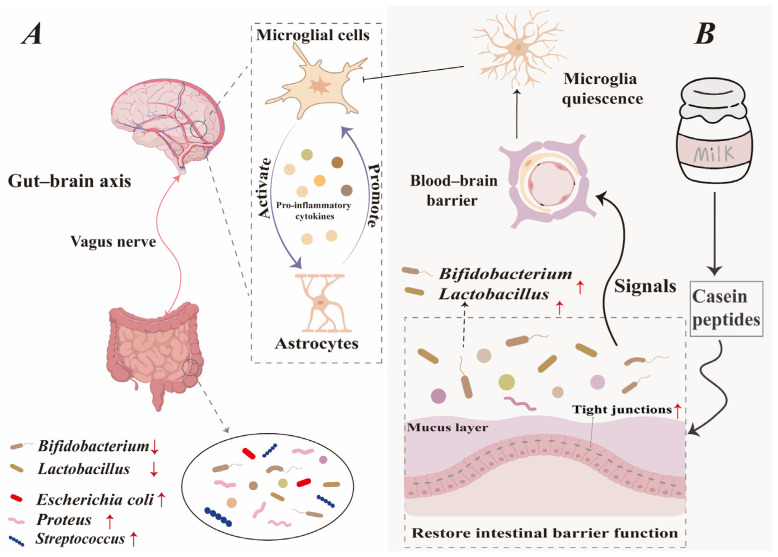
Casein peptides regulate the gut–brain axis to enhance the intestinal barrier and alleviate IBD. (**A**) The disorder of intestinal microbiota generates signals through the gut–brain axis, leading to IBD. (**B**) Casein peptides play a positive regulatory role in the intestinal barrier through the gut–brain axis.

The CNS comprises the brain and spinal cord. The CNS is tasked with taking in sensory data, interpreting and issuing command signals [81]. The blood–brain barrier (BBB) naturally shields the CNS by blocking harmful substances, but this protective function also limits drug delivery and reduces treatment effectiveness for CNS disorders [82]. The BBB’s core structure consists of stellate glial cells [83]. Activated astrocytes heighten inflammatory signals while weakening the TJs between endothelial cells—this dual action compromises the BBB’s protective function [84]. As shown in Figure 4, the gut–brain axis transmits signals generated by gut microbiota dysbiosis in IBD patients to the CNS. This activates microglia, which in turn triggers astrocytes to release inflammatory cytokines [85], further promoting glial cell activation [86]. Casein peptides regulate gut microbiota composition to maintain intestinal homeostasis. Signals of gut stability traverse the BBB via the gut–brain axis, helping sustain glial cells in a quiescent state. This inhibits astrocyte activation and reduces inflammatory responses.

In vitro studies have demonstrated that some foodborne peptides and hormones can cross the BBB [87]. To investigate whether casein peptides possess the potential to cross the BBB, researchers identified 192 peptides from milk. Among these, one casein peptide (PIGSENSEKTTMPLW) was capable of traversing both the intestinal barrier and the BBB model [88]. Although a small number of in vitro experiments have confirmed that casein peptides can cross the BBB model, research in this field remains largely unexplored to date. Future studies may further investigate the trans-BBB properties of casein peptides in vitro, which is expected to provide novel strategies and insights for the targeted treatment of IBD with casein peptides.

## 4. Challenges and Solutions Faced by Casein Peptides

The therapeutic effect of casein peptides against IBD primarily stems from the preservation of intestinal barrier integrity. Compared with drug therapy, casein peptides have many advantages and can open up a new avenue for the management of IBD. However, casein peptides are hindered by problems such as low bioavailability, lack of identification techniques, and scarcity of clinical research. Here, some solutions are also proposed to assist in the research experiments of IBD.

### 4.1. Improve the Bioavailability of Casein Peptide

Oral delivery is the most convenient method for administering bioactive peptides. However, the structural characteristics of casein peptides, combined with the physicochemical challenges they face and the harsh gastrointestinal environment, often lead to their breakdown during digestion [89]. Consequently, these peptides suffer from limited stability and diminished bioavailability. Research has found that an important reason for the short half-life of bioactive peptides is their sensitivity to gastrointestinal proteases such as pepsin, trypsin, and chymotrypsin [90]. The presence of protease cleavage sites on peptides contributes to their reduced stability in the intestinal environment.

Gels, liposomes, emulsions, and microparticles serve as delivery vehicles for bioactive peptides with specific advantages. Casein peptide encapsulation offers distinct advantages over alternative delivery systems by improving peptide stability, enhancing protective effects, and allowing for controlled release [91]. Liposomes are closed, spherical vesicles composed of concentric phospholipid layers stabilized by hydrophobic interactions. This architecture shields encapsulated compounds from enzymatic breakdown and improves their bioavailability in vivo [92].

During a six-day storage period, encapsulation of casein peptide liposomes effectively maintained casein peptide stability, at the same time preserving their structure and function at 4 °C. This approach also positively influenced microbial composition by increasing beneficial bacteria and reducing harmful bacteria, though extended studies are needed to evaluate long-term stability under various conditions [93]. Liposomes show strong potential as a delivery system for encapsulating, stabilizing, and directing casein peptides to specific sites. Research in this area is still limited, and future work should focus on measuring how well these peptides are absorbed in living organisms and whether targeted release can be reliably achieved.

### 4.2. Increase Clinical Research

The study of casein peptides for IBD relies largely upon in vitro and animal experiments in contrast to the limited number of clinical investigations in human subjects. The commonly used models of IBD in animal experiments include transgenic models, gene knockout models, chemical models and adoptive-transfer models. Key models of acute and chronic forms of colitis are induced by 2,4,6-trinitro-benzene sulfonic acid, oxazolone and DSS [94]. In vitro models for intestinal barrier research are classified by complexity into several categories: basic epithelial monolayer models (e.g., Caco-2 monoculture), more physiologically relevant epithelial–goblet cell co-culture models (e.g., Caco-2/HT29-MTX co-culture), and co-culture models incorporating immune cells for simulating immune inflammation (e.g., Caco-2/HT29-MTX/THP-1 tri-culture systems) [95]. The intrinsic ability of Caco-2 cells to differentiate into polarized monolayers enables them to mimic the intestinal barrier with functional properties such as selective permeability and transepithelial electrical resistance [96]. However, as these cells are derived from colorectal cancer, they fail to express typical TJ proteins under inflammatory conditions, which represents a major limitation of in vitro models based on Caco-2 cells. To optimize the in vitro model of the human intestinal epithelial barrier, researcher has found that human intestinal organs can display typical features that occur during inflammation, such as disruption of intercellular adhesion accompanied by the redistribution and depletion of cadherins, leading to a reduction in cell junctions at the cell boundaries and thus triggering intestinal barrier dysfunction [97]. Intestinal organoid culture is broadly recognized as a highly promising model for studying the intestinal barrier. However, its practical adoption for this purpose is hampered by a scarcity of detailed experimental protocols for evaluating barrier properties and changes induced by cytokines [98]. Whether casein peptides can fully exert their functions in the intestines of inflammatory patients still needs to be confirmed by further clinical research. Patients with UC and CD exhibit differences in lesion locations and disease progression, leading to varying treatment responses and outcomes [99]. Consequently, individual variability poses a significant challenge in clinical research. In future clinical studies, patients should be stratified for treatment based on biomarker criteria and combined with precision tools to ensure optimal therapeutic outcomes [100].

### 4.3. Enhance the Identification Technology of Casein Peptides in Regulating the Intestinal Barrier

Traditional bioactive peptide identification follows a multi-stage process from extraction to activity verification, making it time-consuming and susceptible to human factors that limit efficiency. Modern techniques involving bioinformatics, molecular docking, and molecular dynamics simulation, which provide efficient approaches for the rapid screening and evaluation of peptides, are widely applied in peptide-related research [101]. Bioinformatics techniques, such as computer simulation screening, quantitative structure–activity relationships, and machine learning, have become indispensable tools in the study of bioactive peptides [102]. Integrated computational platforms combining hybrid deep learning with traditional machine learning are advancing bioactive peptide identification through optimized algorithms and enhanced analytical accuracy [103]. The combination of network pharmacology and machine learning allows for efficient screening and prioritization of potential bioactive peptides. Combining hierarchical statistical mechanical modeling (HSM) [104] and a deep learning framework for multi-level peptide-protein interaction prediction (CAMP) [105] in computer methods can refine the precision of peptide prediction, thereby facilitating the identification of targets and pathways of peptides [106]. Following an initial screening of potential peptides by the HSM model, the CAMP model narrows down the selection through computational modeling of protein–peptide complexes [107]. Using bioinformatics techniques, two novel yak-casein-derived peptides, QEPVLGPVRGPPPP and VYPFPPPN, were identified. These peptides enhance the survival rate of RAW 264.7 macrophages and inhibit the release of NO, TNF-α, and IL-6, indicating that both peptides identified through bioinformatics possess safe anti-inflammatory potential [108]. Bioinformatics techniques improve the screening and functional prediction of casein peptides, yet their application in intestinal barrier research remains at an early stage without a standardized validation system. Future work must focus on developing more compatible algorithms, clearer models, and stronger experimental confirmation.

## 5. Conclusions and Prospects

Current biologic and drug treatments for IBD are often costly and carry side effects, motivating researchers to explore safer options. Milk-derived casein peptides offer a naturally safe and promising alternative in this search. This paper systematically reviews the structure of the intestinal barrier, discusses the impact of intestinal barrier damage on IBD, and highlights the potential role of casein peptides in alleviating IBD by maintaining and repairing the intestinal barrier through multi-targeted, multi-level mechanisms.

This study explored casein peptides’ effects on gut barrier function in IBD, particularly regarding microbiome. IBD origins require further investigation, but current proof indicates gut flora modification holds therapeutic promise. Microbes significantly aid damaged barrier recovery, confirming barrier impairment as an early IBD pathology. Comparative data reveal that IBD patients have decreased MUC2 expression, disorganized TJs and altered gut microbiota versus healthy individuals, all of which demonstrate intestinal barrier compromise. The proven barrier–disease connection supports barrier-focused treatment strategies.

Moreover, by analyzing several mechanisms through which casein peptides regulate the intestinal barrier, we believe that casein peptides repair the intestinal barrier via multi-targeted action. Gut microbiota play a crucial role in regulating the intestinal barrier through casein peptides. Metabolic products of the gut microbiota, such as short-chain fatty acids, can stimulate intestinal motility. Changes in the composition of the gut microbiome may also activate signaling pathways, thereby releasing anti-inflammatory factors or influencing macrophages, thus enhancing the body’s immune system. Within the gut–brain axis regulation, gut microbiota release signaling molecules that transmit signals to vagus nerve endings, which in turn relay them to the CNS. The CNS then initiates reciprocal regulation by secreting key factors, accordingly influencing gut health. Through synergistic interactions on multiple targets, casein peptides promote more efficient intestinal barrier restoration, contributing to the amelioration of IBD. Exploring the relationship of the gut–brain axis, it was found that the intestinal system and the nervous system act in both directions, opening up a new pathway for casein peptides to repair the intestinal barrier. However, casein peptides are difficult to identify due to their complex structure. Traditional extraction, purification and mass spectrometry identification methods are time-consuming and labor-intensive. Bioinformatics techniques provide a convenient approach for the structural characterization of casein peptides. Here, we mentioned the coupling techniques for the identification of bioactive peptides, such as combining network pharmacology with machine learning. Current analytical methods allow exact characterization of casein peptides including their cellular targets and functional roles.

Casein peptides face multiple challenges in the gut, including enzymatic breakdown, a variable gastrointestinal environment, and microbe interactions. These factors damage peptide structures and limit their benefits. Advanced delivery systems solve these problems by providing protection and targeted release. New research shows that better carrier designs and combined methods greatly improve peptide absorption and function. The current understanding of how casein peptides protect gut barriers and reduce IBD symptoms comes mainly from cell and animal studies. Whether these promising results will apply to human patients needs to be confirmed through proper clinical trials. By elucidating their regulatory effects on the intestinal barrier, this research offers a highly promising therapeutic strategy for IBD. Future clinical studies are needed to validate these findings and support the translation of casein peptides into further applications.

## Figures and Tables

**Table 1:** Functional properties of casein-derived peptide mixtures and proposed regulatory mechanisms.

No.	Mechanism	Peptide Sequence	Active Effect	References
1	Regulate the composition of microorganisms	The casein peptide mixture contains peptide FFVAPFPEVFGK	Increased levels of anti-inflammatory gut microbes *Roseburia lenta*, *Agathobacillus butyriciproducens*, and *Adlercreutzia equolifaciens*.	[35]
2		Dietary β-casein peptide QEPVL	Enhanced *Bacteroidaceae* and *Firmicutes*, especially *Erysipelotrichaceae*, to recover the intestinal microbiome.	[36]
3	Microbial metabolites	The casein peptide mixture contains peptide FFVAPFPEVFGK	Increased short-chain fatty acid content.	[35]
4	Enhance mucus integrity	Bovine β-casein fragment (108–113)	Increased MUC4 expression, while MUC2 and MUC5AC levels remained unchanged.	[37]
5	Immune regulation	The mixture contains peptide DQPFFHYN and YSPFSSFPR	Inhibited the secretion of pro-inflammatory cytokine NO, TNF-α and IL-6 secretion, and suppressed the expression of iNOS in RAW264.7 cells.	[38]
6		β-casein derived peptide QEPVL	Inhibited the production of pro-inflammatory cytokines, TNF-α, and IFN-γ, while increasing IL-10 levels.	[39]
7		Casein derived peptide PFPEVFG	Significantly reduced IL-6, TNF-α and NO expression.	[40]
8		Buffalo casein derived decapeptide YQEPVLGPVR	Reduced IFN-γ levels, coupled with elevated IL-10 and TGF-β levels, indicated an anti-inflammatory response.	[41]
9	Activate the signaling pathway	Casein derived peptide AMKPWIQPK	Activated the MAPKs/NF-κB signaling pathway.	[42]
10		β-casein derived peptide CASB_135–150_	Inhibited NF-κB signaling and reduced inflammation.	[43]
11		Milk-derived β-casein peptide VKEAMAPK	Bound to the Keap1 protein and activated the Nrf2 pathway.	[44]
12		Casein phosphopeptide EIVPNSpAEER	Inhibited the two signaling pathways of CaSR/PI3K/NF-κB and CaSR/PLC/NF-κB.	[45]
13	Upregulate tight-junction proteins	Casein phosphopeptide EIVPNSpAEER	Increased the expression of tight-junction proteins claudin-1, occludin and ZO-1.	[45]

## Data Availability

No new data were created or analyzed in this study. Data sharing is not applicable to this article.
